# ‘There’s something about admitting that you are lonely’ – prevalence, impact and solutions to loneliness in terminal illness: An explanatory sequential multi-methods study

**DOI:** 10.1177/02692163221122269

**Published:** 2022-09-08

**Authors:** Jeffrey R Hanna, Tracey McConnell, Craig Harrison, Katarzyna A Patynowska, Anne M Finucane, Briony Hudson, Sharon Paradine, Angela McCullagh, Joanne Reid

**Affiliations:** 1 Queen’s University Belfast, Belfast, UK; 2 Marie Curie Hospice Belfast, Belfast, UK; 3 Marie Curie Hospice Edinburgh, Edinburgh, UK; 4 Marie Curie, London, UK

**Keywords:** Loneliness, terminal illness, carers, end of life, palliative care, healthcare professionals, social care professionals

## Abstract

**Background:**

Loneliness is a prevalent societal issue and can impact on a person’s physical and mental health. It is unclear how loneliness impacts on end of life experiences or how such feelings can be alleviated.

**Aim:**

To explore the perceived prevalence, impact and possible solutions to loneliness among people who are terminally ill and their carers in Northern Ireland through the lens of health and social care professionals.

**Design:**

An explanatory multi-method study.

**Setting/participants:**

An online survey (*n* = 68, response rate 30%) followed by three online focus groups with palliative and end of life care health and social care professionals (*n* = 14). Data were analysed using descriptive statistics and thematic analysis.

**Results:**

Loneliness was perceived by professionals as highly prevalent for people with a terminal illness (92.6%) and their carers (86.8%). Loneliness was considered a taboo subject and impacts on symptoms including pain and breathlessness and overall wellbeing at end of life. Social support was viewed as central towards alleviating feelings of loneliness and promoting connectedness at end of life. Four themes were identified: (1) the stigma of loneliness, (2) COVID-19: The loneliness pandemic (3) impact of loneliness across physical and mental health domains and (4) the power of social networks.

**Conclusion:**

There is a need for greater investment for social support initiatives to tackle experiences of loneliness at end of life. These services must be co-produced with people impacted by terminal illness to ensure they meet the needs of this population.

**What is already known about the topic?**

- Experiences of loneliness are heightened for people impacted by terminal illness.
- Individuals who experience loneliness are at risk of a variety of physical and mental conditions including heart disease, obesity, a weakened immune system, anxiety or depression.
- Loneliness is a global public health issue that has become more prevalent during the COVID-19 pandemic.

**What this paper adds?**

- Loneliness is perceived by healthcare professionals as a common experience for patients and carers at the end of life which has become more commonplace during the COVID-19 pandemic.
- The stigma around admitting to feeling lonely among those impacted by terminal illness makes it difficult for others to identify.
- Loneliness impacts on physical and mental health symptoms experienced at end of life including pain, breathlessness or feeling there is nothing left to live for.

**Implications for practice, theory, or policy**

- A public health approach may help raise awareness of loneliness as a serious issue at end of life and help combat the associated stigma.
- Social support initiatives that offer opportunities for social connectedness for people with a terminal illness, and their families/carers are needed to alleviate experiences of loneliness.
- Potential solutions to loneliness require further research with those directly impacted by terminal illness: patients and their caregivers.

## Introduction

Loneliness is defined as a subjective, unwelcome feeling of lack or loss of companionship that happens when there is a mismatch between the quantity and quality of social relationships that we have and those that we want.^1^ It is estimated that 15% of adults aged 16–79 in the United Kingdom report high levels of loneliness in their daily lives, with this number doubling in those aged 80 plus.^2^ People in later life, or those with ill-health, a disability or low income are at risk of experiencing loneliness.^3,4^ For people with terminal illnesses, strong feelings of loneliness have been reported in studies internationally.^5,6^

A recent systematic review drawing on international literature identified associations between providing informal care and higher loneliness levels.^7^ In the UK, it is estimated 8-in-10 informal carers have felt lonely or socially isolated as a result of their caring situation.^8^ An informal carer (hereafter referred to as ‘carer’ for simplicity) is defined as a person who provides unpaid care to someone important to them because of long-term physical or mental health, disability or issues related to age.^9^ Prevalence rates of loneliness among carers have soared during the COVID-19 pandemic in regions such as the UK, USA, Ireland and New Zealand.^10^ Although feelings of loneliness are heightened for people impacted by terminal illness,^11,12^ it is not clear what impact the pandemic has had on these experiences.

Loneliness can have a detrimental impact on a person’s physical^13,14^ and mental health.^15–17^ Their existential health, defined as finding meaning and purpose in life and developing supportive social relationships, can also be severely impacted.^18^ Carers who describe experiences of loneliness have higher rates of stress and report lower levels of wellbeing than the general population,^19^ as well as greater risk of developing illness.^20^ However, it remains unclear how experiences of loneliness impact the health and wellbeing of people with a terminal illness.

There is a need to understand how experiences of loneliness can be mitigated for this vulnerable population; especially given estimations that the number of people living with a terminal illness in the United Kingdom is expected to increase up to 42% by 2040,^21^ and the need for informal carers up to 40% by 2037.^9^ A better understanding of the prevalence, impact and possible solutions of loneliness for people with a terminal illness and their carers pre- and during the COVID-19 pandemic can aid our understanding of how best to alleviate such experiences and promote better end of life experiences. For the purpose of this study, a person with a terminal illness is someone who is not on a curative pathway for their illness. This includes the end of life period, often characterised as the final 12 months of life.^22^

## Research questions

(1) What is the perceived prevalence of loneliness among people who are terminally ill and their carers?
(2) What is the perceived impact of loneliness among people who are terminally ill and their carers?
(3) What are the possible solutions to alleviating loneliness among people who are terminally ill and their carers?

## Methods

### Design

An explanatory sequential multi-methods design was used. This design starts with the collection and analysis of quantitative data, followed by the collection and analysis of qualitative data in order to interpret or expand on the first-phase quantitative results.^23^Our study consisted of an online closed survey for Marie Curie Northern Ireland health and social care professionals to determine the perceived prevalence, impact and possible solutions to loneliness, followed by focus groups to further explore, elaborate on and understand the survey findings.^24^

### Setting

This study was conducted within Marie Curie Northern Ireland community nursing services which serves an overall population of over 1.8 million. Services include hospice at home, rapid response and outpatient services (i.e. Day Hospice).

### Patient and public involvement

Two members of the Marie Curie Research Voices (AM, SP) were involved throughout the research project, from shaping the grant application to dissemination of findings. Themes from analysis were also refined following feedback from established Patient and Public Involvement groups including four members of the Marie Curie Research Voices and two members of the All Ireland Institute of Hospice and Palliative Care Voices4Care groups.

### Study population

Individuals were considered eligible if they were a member of the Marie Curie Northern Ireland community nursing service (registered nurses and healthcare assistants), members of the patient and family support team made up of a bereavement coordinator, chaplain and social workers, as well as Day Hospice and rehabilitation team staff (hereafter referred to as ‘professionals’ for simplicity).

### Sampling

Using convenience sampling for the survey and purposive sampling for the focus groups a range of professionals were recruited to the study. At the time of recruitment, 225 professionals were eligible to take part in the survey and focus groups.

### Recruitment

#### Survey

An invitation to complete the survey was circulated to eligible individuals (outlined under Study Population) via email by the nursing service, Day Hospice, patient and family support team and rehabilitation team lead. The email provided individuals with the study information. Presumed consent was used for anonymous participation and the following statement was included at the start of the survey ‘by completing this survey, you are consenting to participate in this study’.

#### Focus groups

Individuals invited to participate in the survey were also invited via email by team leads within the recruiting institution to take part in a focus group. Participant information sheets were attached to emails outlining the purpose of the study, the processes involved with taking part, possible risks and benefits, their rights, privacy and confidentiality and whom to contact if they had further questions or were still interested to take part. Interested professionals completed an online consent form before the focus group.

### Data collection

An online survey was developed using Microsoft Forms. Survey questions were developed by the research team and Patient and Public Involvement representatives involved in the study. Initially participants were asked demographic questions about their gender, ethnicity and job role. Respondents were then asked open-ended and closed questions about the perceived prevalence and impact of loneliness for people living with a terminal illness and their carers, and possible solutions to alleviate these experiences. As the study was conducted during the COVID-19 pandemic, specific questions were asked about the perceived impact and prevalence of loneliness for those affected by terminal illness pre and during the pandemic (see supplementary file for the questionnaire). The survey was open between June and July 2021.

Three focus groups were conducted between September and October 2021. A topic guide (Table 1) was developed by the research team based on the study aims and objectives and findings from the survey. The guide was refined through feedback by the Patient and Public Involvement representatives. Focus groups were led by the second author (TMcC) and facilitated by two researchers (JR, KP). TMcC, JR and KP are female researchers with research and clinical experiences of working with people impacted by terminal illness and conducting qualitative research. TMcC and KP were known to some of the participants through their roles as Senior Research Fellow and Research Nurse at the recruiting institution. The use of reflexive notes, peer debriefing and data source triangulation were applied to mitigate bias.^25^ The focus groups were conducted and recorded on Microsoft Teams and lasted up to 60 min (range 50–60 min).

**Table 1.** Semi-structured topic guide used to guide the conduct of the study.

Sample of topics based on the quantitative survey findings and study aims and objectives - Exploration as to why professionals are more likely to observe loneliness rather than individuals themselves reporting this. - Exploration as to how loneliness impacts on a person’s overall health and wellbeing during terminal illness. - Perceptions as to how professionals can better identify loneliness during terminal illness. - Perceived obstacles to reducing loneliness in terminal illness. - Perceptions as to how loneliness could be alleviated for patients and carers.

### Data analysis

Data were analysed in three phases reflecting the explanatory sequential design: (1) analysis of quantitative data, (2) analysis of qualitative data and (3) analysis of how the qualitative data helps explain the quantitative data to answer the research questions.

Quantitative data were analysed using descriptive statistics within Microsoft Excel by JRH and TMcC. Qualitative open text responses were independently analysed into core themes by four members of the research team (TMcC, JR, AMF, BFH) with feedback from Patient and Public Involvement members (AM, SP) determining the final themes. These core themes shaped the subsequent focus group schedule.

Focus group Microsoft Teams recordings were transcribed *verbatim* by an external transcription service provider. Qualitative data from focus groups were analysed using reflexive thematic analysis.^26^ This was led by the first author (JRH), a post-doctoral male researcher and nursing student with experience of conducting and analysing qualitative studies in end of life care. First, transcripts were read to gain a sense of the participants’ experiences and perceptions. Data were then coded and managed using NVIVO v.12 by marking similar phrases and words in the narratives related to the study questions. Using manual mind-mapping techniques, JRH identified where the codes merged to form themes and sub-themes. Transcripts and themes were independently analysed by three co-authors (TMcC, JR, CH) and refined through critical dialogue with all authors. Themes were further reviewed and refined by feedback from Patient and Public Involvement representatives.

### Ethical considerations

Participants were provided with detailed written information about the purpose of the research. Written consent was obtained prior to taking part in the focus group. Participants were informed of their right to withdraw from the online survey or focus groups at any point without negative impact. However, participants were informed that any data already collected during online focus groups prior to their withdrawal would be retained for the study. Data protection procedures were observed and assurances of confidentiality were given. Pseudonyms have been adopted in the results section of this paper to maintain confidentiality. Ethical approval was obtained from Queen’s University Belfast (REF code: MHLS 21_58) and Research Governance approval was obtained from Marie Curie Northern Ireland (Ref code: MCNI 07.21).

## Results

### Quantitative survey findings

A total of 68 (response rate 30%) professionals completed the survey, of which 66 were female, and had at least 1-year experience of providing end of life care (Avg = 13.7 years, range 1–37 years). Respondents included nurses (*n* = 42), healthcare assistants (*n* = 16), social care and allied health professionals (*n* = 10).

### Perceived prevalence of loneliness among people who are terminally ill and their carers

92.6% (63/68) of respondents reported perceived loneliness among their patients pre-pandemic. Also, 86.8% (59/68) of respondents felt they had supported carers experiencing loneliness pre-pandemic. A high proportion, 72.1% (49/68) of respondents felt there was a substantial increase in loneliness among people with a terminal illness and carers 69.1% (47/68) during the COVID-19 pandemic (Table 2).

**Table 2.** Survey responses from professionals (*n* = 68).

Variable	*n* (%)
*Question: In your opinion/ from your perspective to what extent has the COVID-19 pandemic impacted on the prevalence of loneliness among the palliative and end of life care patients you care for?*	
No increase in the numbers experiencing loneliness	1 (1.5)
Slight increase in the numbers experiencing loneliness	6 (8.8)
Moderate increase in the numbers experiencing loneliness	12 (17.6)
Substantial increase in the numbers experiencing loneliness	49 (72.1)
*Question: In your opinion/from your perspective to what extent has the COVID-19 pandemic impacted on the prevalence of loneliness among their informal carers?*	
No increase in the numbers experiencing loneliness	1 (1.5)
Slight increase in the numbers experiencing loneliness	4 (5.9)
Moderate increase in the numbers experiencing loneliness	16 (23.5)
Substantial increase in the numbers experiencing loneliness	47 (69.1)

While survey respondents stated they were aware someone in their care was experiencing loneliness at end of life, this conclusion was often based on their own observations of the patient’s/carers’ circumstances in 88.2% (60/68) of cases, rather than from self-report by the carer (42.7%, 29/68) or the patient (55.9%, 38/68) (Table 3).

**Table 3.** Survey responses from professionals.

Variable	*n* (%)
*Question: What caused you to conclude that the person was experiencing loneliness? Select as many as appropriate.* (Please skip this question if not applicable)	
My own observations about the patients’ circumstances	60 (88.2)
The patient told me/indicated that the patient was experiencing loneliness	38 (55.9)
Informal carer told me / indicated that the patient	29 (42.3)
Issue never raised by patients or carers and no obvious signs	1 (1.5%)
Not something I would formally assess.	1 (1.5%)
Low mood, no purpose to life, no one to contact	1 (1.5)
They have had the same good family support throughout	1 (1.5)

### Perceived impact of loneliness among people who are terminally ill and their carers

Most respondents rated loneliness as having a high impact on patient’s psychological (63.2%, 43/68) and social (55.9%, 38/68) wellbeing and carers psychological (66.2%, 45/68) and social (60.3%, 41/68) wellbeing (Table 4).

**Table 4.** Survey responses from professionals.

Variable	High *n* (%)	Medium *n* (%)	Low *n* (%)	None *n* (%)
*Question: How would you rate the impact of loneliness of the palliative and end of life care experiences of the patients you cared for or supported?*				
Psychological wellbeing	43 (63.2)	19 (27.9)	5 (7.4)	1 (1.5)
Spiritual wellbeing	26 (38.2)	35 (51.5)	5 (7.4)	2 (2.9)
Physical wellbeing	22 (32.4)	38 (55.9)	6 (8.8)	2 (2.9)
Social wellbeing	38(55.9)	25 (36.7)	4 (5.9)	1 (1.5)
*Question: How would you rate the impact of loneliness on the informal carers you supported?*				
Psychological wellbeing	45 (66.2)	13 (19.1)	9 (13.2)	1 (1.5)
Spiritual wellbeing	28 (41.2)	28 (41.2)	11 (16.1)	1 (1.5)
Physical wellbeing	27 (39.7)	32 (47.0)	8 (11.8)	1 (1.5)
Social wellbeing	41 (60.3)	17 (25.0)	9 (13.2)	1 (1.5)

### Qualitative findings

Fourteen female professionals then took part in one of the three focus groups. Participants included nurses (*n* = 3), healthcare assistants (*n* = 2), social care (*n* = 5) and allied health professionals (*n* = 4). Findings are discussed under three sections and corresponding themes to address the research questions on prevalence, impact and possible solutions to alleviating loneliness among people who are terminally ill and their carers:

(1) Explaining the prevalence of loneliness
  Themes: (1) the stigma of loneliness and (2) COVID-19: The loneliness pandemic
(2) The impact of loneliness
  Theme: (3) impact of loneliness across physical and mental health domains
(3) Possible solutions to loneliness
  Theme: (4) the power of social networks.

#### Theme 1: The stigma of loneliness

Professionals believed loneliness is a highly prevalent issue for people with a terminal illness and their carers, and considered such experiences are caused by a lack of available support networks in the person’s life to provide emotional and practical support throughout the end of life period. Another reported factor included carers’ lack of time to maximise their networks due to the demands of the caring role. However, professionals believed loneliness is a seldom disclosed issue at end of life for patients and their carers and felt these populations ‘*suffer in silenc*e’. Often loneliness was perceived as an *‘invisible issue’* and challenging for professionals to identify.

*‘There’s something about admitting that you are lonely. It’s almost as if there’s a stigma around loneliness. With loneliness it kind of comes under that mental health bracket or umbrella, and there’s no. . . either it’s taken as a sign of weakness or some sort of stigma around that, and there’s no quick fix’. [Mabel, Allied Health Professional]*

*‘One lady was cared for by her husband. She had a degenerative condition that had been ongoing for a while. And she actually said to me “this is the first time I’ve been able to talk to another woman about how I feel”. And it broke my heart, when you think of the amount of people coming in and out of the house. But it was just because I was there and I was there all night. And she was awake most of the night. And I think the impact for her was, she felt very sad. She felt she couldn’t tell anybody how it was, how she felt. Not even her husband, because she was protecting him. She was lonely. Bottom line was, she was lonely’. [Florence, Nurse]*

While loneliness was discussed as a historical and ongoing issue for people with a terminal illness and their carers, participants described heightened experiences of loneliness during the COVID-19 pandemic.

#### Theme 2: COVID-19: The loneliness pandemic

Professionals spoke of how outpatients had experienced a heightened sense of loneliness caused by the pandemic. While professionals recognised the impact of the pandemic and increased loneliness among people in general, they emphasised one key difference in relation to their patients, and that is the terminal nature of their conditions, which meant the heightened loneliness they experienced due to the pandemic would be their final experience at their end of their life.

*‘And [I remember] the occupational therapist going out and one very elderly lady saying to her, you’re the first person who has sat on that sofa for 12 months. . . and that lady has since died’. [Patricia, Nurse]*

Carers were viewed by professionals as having reduced opportunities to maximise their social networks during the pandemic. Professionals reported reduced availability of services such as those provided by Day Hospice or community groups, which were described as providing vital support networks pre-pandemic for carers and people with a terminal illness to meet others who were experiencing similar situations to themselves, coined as peer-support. Participants noted how some services moved to virtual platforms during the pandemic and were beneficial for people living in rural areas who would normally be too far to attend in person. However, the online format was only appropriate for those with internet access.

*‘Everyone says how much they miss the Day Hospice. Just the sense of being able to come in, to see any professionals they needed to see, but also to catch up with other patients who have become friends. And for the carers to be able. . . their loved ones to be able to talk to each other. And just the general sense of fun, almost. Yeah, people really enjoyed that sense of being together and got a lot from it’. [Jean, Social Care Professional]*

#### Theme 3: Impact of loneliness across physical and mental health domains

Professionals often felt experiences of loneliness at end of life has a *‘ripple effect’* on a person’s mental and physical health. Moreover, it was perceived that when patients and carers avoid talking about their experiences of loneliness this has a negative impact on the person’s wellbeing and quality of life. Professionals believed loneliness led to patients feeling like *‘giving up’* as they felt they had nothing to continue living for.

*‘Loneliness, if not dealt with or discussed, usually leads to giving up on life. Like there’s nothing to care about or to live for’. [Sandra, Nurse]*

More often, professionals perceived patients’ experiences of loneliness at end of life impacts on a person’s symptoms ‘*especially around pain, breathlessness and anxiety’*, with an increased need for medications or involvement with healthcare services which were described by participants as ‘*already stretched’*.

*‘I was working last night in rapid response, and Tony has been on the system three times. I have been out to him the last two weeks, nearly every night that I’ve been working. And the issue is not his catheter. The issue is that he is lonely. . . he has no family here. His nearest family is in England. His mood is quite low as well. He says he has nobody to talk to. And he’s ringing out of hours to get me out, or get one of us out, to come and talk to him. . . He is isolated and lonely in his home. And people have not picked up on that’. [Freda, Nurse]*

#### Theme 4: The power of social networks

Professionals recognised the importance of social networks for people with a terminal illness and their carers. However, professionals discussed how this is not always possible due to a lack of social networks in some people’s lives, as well as factors such as people living too far away from others, family members being estranged to the carer or person with the terminal illness, or people’s lack of time to help due to the busyness of their own lives.

Participants felt Day Hospice was helpful to alleviate feelings of loneliness for patients and carers at end of life. It was believed Day Hospice provided patients and carers with peer-support opportunities, including meeting, sharing and learning from others who are experiencing similar situations to themselves in a face-to-face environment. Also, Day Hospice was considered as helpful to developing *‘genuine friendships’*, further enhancing a person’s social support network pre (and post for carers) death and developing a sense of community.

*‘I think that’s what’s so brilliant about the Day Hospice. . . it starts off as being part of their medical care. And then it transforms into this wonderful social opportunity, and well holistic is a good word for it. It caters for all needs, once you get the people through the door. And they are glad to come because it’s part of their. . . it starts off as part of their treatment. And then blossoms’. [Jean, Social Care Professional]*

Other participants considered how Day Hospice services provided carers with opportunities for respite and to engage in social activities outside of the caregiving role and maintain some sense of normality. However, professionals feared that these *‘softer measures’* of benefit to alleviating loneliness would not convince those in power making the decisions that this is a model that necessitates investment.

*‘. . .And the need for maybe respite support. So that they are maybe getting two afternoons a week that they can go and do a hair appointment if they need to. Attend to their own needs with the GP. Go for a walk with a friend. It’s just trying to not only focus on the patient’s needs but identify the impact of emotional and mental wellbeing and loneliness and isolation in a caring role’. [Margaret, Social Care Professional]*

*‘But it’s hard to quantify that, isn’t it? So therefore, we don’t have the stats and the figures to show those in power’ [Harriet, Social Care Professional]*

There were some participants that considered how the availability of community projects such as walking groups, community projects such as planting pots of flowers, or coffee mornings in local community centres could help to promote peer-support and respite opportunities for people with a terminal illness and their carers, and reduce experiences of loneliness at end of life. However, participants felt that these may be less well attended by people who feel lonely as they may perceive they are not *‘worthy’* or consider it ‘*embarrassing’* to attend alone.

*‘I suppose I’m just thinking, community initiatives and everything, it’s fantastic. But for some people, whenever they get to the point of being so lonely that their self-esteem has been impacted by it, they are not going to go to anything that is available, because at that point they may not even feel worthy of being a member of a group or whatever. If someone’s mental health is so badly affected, then they are more likely to not avail of what is there’ [Anne, Social Care Professional]*

## Discussion

Loneliness was reported by healthcare professionals as prevalent among people with a terminal illness and their carers. While studies have highlighted loneliness as a prevalent issue during COVID-19 due to restrictions to protect public health,^27–29^ findings highlight such experiences were exacerbated for carers (69.1%) and people with a terminal illness (72.1%) as they approached end of life.

Loneliness at end of life was described as having a detrimental impact on a person’s health and wellbeing, including worsening end of life physical symptoms such as breathlessness and pain, and a range of mental health symptoms such as anxiety and depression. Similar findings have been reported in the literature with other populations such as people in later life and those with cancer.^30–32^ Significantly, loneliness was also linked with existential suffering, and professionals felt experiences of loneliness at end of life may lead patients to feel that they have nothing left to live for. The need to be ‘present’ with others, and to have a sense of ‘connectedness’ is closely linked with existential loneliness.^33^ Despite reduced opportunities, people with an advanced illness and their carers have a desire for social connection. Connection with healthcare professionals, and the ‘presence’ of others carries significant spiritual and existential meanings for terminally ill people.^34^ There is a need to alleviate existential concerns to promote better end of life experiences as well as adjustment in the post-death period for carers. Sensitivity and a trusting relationship between the healthcare professional and the person with a terminal illness, alongside openness and awareness of the person’s concerns provides a good basis for discussion of existential concerns.^35^ Failure to address experiences of loneliness at end of life may have ramifications for pressured health and social care services such as an increased need for services.^36,37^

Findings highlighted challenges in identifying experiences of loneliness in people with a terminal illness and their carers due to the taboo nature of admitting feeling lonely. Professionals in this study appeared to be central to identifying loneliness in those impacted by terminal illness. This highlights the importance of communication skills training for new staff to support discussions around experiences of loneliness,^38,39^ along with further research on when to start such conversations, so that professionals are appropriately skilled in identifying experiences of loneliness in people impacted by terminal illness. It could be argued a public health approach is necessary to raise awareness of loneliness as a serious issue at end of life, combat stigma and ensure those who have been suffering in silence get the support they need.^40^

It appeared that social and emotional connectedness with others is helpful to alleviating experiences of loneliness for people with a terminal illness and their carers.^41^ Described in the findings as indirectly facilitated through the ‘Day Hospice Model’, this includes services that provide rehabilitation programmes or complementary therapies.^42^ Societal shifts such as families living further away from each other or communities becoming more fluid as a consequence of people ‘moving about’ due to lifestyle and work-related factors,^43^ a person’s social networks may not be able to provide them with practical and emotional support required at end of life. This may explain why peer-support and creating opportunities to meet others was consistently reported by professionals as a key solution to addressing loneliness for people with a terminal illness and their carers. Other factors included the need to be with people experiencing similar situations who will better understand the person’s circumstances,^44^ or not wanting to feel like a burden to loved ones.^45^

Peer-support is helpful to promoting better mental and physical outcomes for individuals, for example through befriending and companionship services.^46^ A social prescribing model also may be helpful for alleviating experiences of loneliness, which could in turn ease the pressures facing health services from increased physical and mental ill health.^47,48^ However, whatever social support approach is taken, it should be co-produced with these populations to ensure it meets their needs. Future research should evaluate the effectiveness of social-support interventions on alleviating experiences of loneliness during terminal illness with a health economics component to demonstrate potential health service savings.^49^

Although other regions of the United Kingdom have national strategies to help address social isolation and loneliness,^50^ Northern Ireland lags as the only region of the UK without such a strategy. The importance of developing this is reflected in the findings from this study, which highlight the high prevalence and impact of loneliness on those who are affected by terminal illness. Such a strategy could provide targeted funding for a public health approach to address loneliness, including groups who are particularly at risk such as those at the end of their lives and their carers.^51^

### Strengths and limitations of the study

This study is largely reflective of the experiences and perceptions of female professionals working within specialist palliative care. Future research could explore professionals’ perceptions of loneliness at end of life in other health and social care settings. Although Patient and Public Involvement representatives were involved in the design, conduct and dissemination of this research, there is a need to explore the impact and possible solutions to loneliness from the perspective of people living with a terminal illness and their carers. We were not able to tease out data to explore how different types of human contact or quality of relationships may impact on loneliness. However, this as an important consideration for future research.

## Conclusion

Overall, findings indicate loneliness is a highly prevalent issue at end of life which has increased as a result of the COVID-19 pandemic. Conversations around loneliness, perhaps via a public health approach are needed to break down the stigma associated with such experiences so those impacted by terminal illness are not suffering in silence. Social connections are imperative, and appear to work best via peer support opportunities, befriending and companionship initiatives. Given the devastating impact of loneliness on quality of life and the wider health service, and the fact there is only one chance to get care right at the end of life, potential solutions to loneliness require further research with those directly impacted: patients and their caregivers.

## Supplemental Material

sj-pdf-1-pmj-10.1177_02692163221122269 – Supplemental material for ‘There’s something about admitting that you are lonely’ – prevalence, impact and solutions to loneliness in terminal illness: An explanatory sequential multi-methods study Supplemental material, sj-pdf-1-pmj-10.1177_02692163221122269 for ‘There’s something about admitting that you are lonely’ – prevalence, impact and solutions to loneliness in terminal illness: An explanatory sequential multi-methods study by Jeffrey R Hanna, Tracey McConnell, Craig Harrison, Katarzyna A Patynowska, Anne M Finucane, Briony Hudson, Sharon Paradine, Angela McCullagh and Joanne Reid in Palliative Medicine

## References

[1] Perlman D Peplau LA . Towards a social psychology of loneliness. In: Duck S Gilmour R (eds) Personal relationships in disorder. London: Academic Press, 1981, pp.32–56.

[2] Thomas J . Insights into loneliness, older people and well-being. London: Office for National Statistics, 2015. https://backup.ons.gov.uk/wp-content/uploads/sites/3/2015/10/Insights-into-Loneliness-Older-People-and-Well-being-2015.pdf (accessed 4 February 2022).

[3] Lim MH Eres R Vasan S . Understanding loneliness in the twenty-first century: an update on correlates, risk factors, and potential solutions. Soc Psychiatry Psychiatr Epidemiol 2020; 55(7): 793–810. 32524169 10.1007/s00127-020-01889-7

[4] Shovestul B Han J Germine L , et al. Risk factors for loneliness: the high relative importance of age versus other factors. PLoS One 2020; 15(2): e0229087. 10.1371/journal.pone.0229087 PMC7012443 32045467

[5] Çıracı Y Nural N Saltürk Z . Loneliness of oncology patients at the end of life. Support Care Cancer 2016; 24(8): 3525–3531. 27007284 10.1007/s00520-016-3159-5

[6] Sand L Strang P . Existential loneliness in a palliative home care setting. J Palliat Med 2006; 9: 1376–1387. 17187546 10.1089/jpm.2006.9.1376

[7] Hajek A Kretzler B König HH . Informal caregiving, loneliness and social isolation: A systematic review. Int J Environ Res Public Health 2021; 18(22): 12101. 34831857 10.3390/ijerph182212101 PMC8618455

[8] Carers UK. Alone and caring: isolation, loneliness and the impact of caring on relationships, http://www.carersuk.org/for-professionals/policy/policy-library/alone-caring (2015, accessed 12 January 2022).

[9] Carers UK . Facts about carers: policy briefing, https://www.carersuk.org/for-professionals/policy/policy-library/facts-about-carers-2015 (2015, accessed 12 January 2022).

[10] Grycuk E Chen Y Almirall-Sanchez A , et al. Care burden, loneliness, and social isolation in caregivers of people with physical and brain health conditions in English-speaking regions: before and during the COVID-19 pandemic. Int J Geriatr Psychiatry 2022; 37(6): 10.1002/gps.5734. 10.1002/gps.5734 PMC9324775 35574817

[11] Rokach A Matalon R Safarov A , et al. The loneliness experience of the dying and of those who care for them. Palliat Support Care 2007; 5(2): 153–159. 17578066 10.1017/s1478951507070228

[12] Vasileiou K Barnett J Barreto M , et al. Experiences of loneliness associated with being an informal caregiver: a qualitative investigation. Front Psychol 2017; 8: 585. 28469589 10.3389/fpsyg.2017.00585 PMC5395647

[13] Valtorta NK Kanaan M Gilbody S , et al. Loneliness and social isolation as risk factors for coronary heart disease and stroke: systematic review and meta-analysis of longitudinal observational studies. Heart 2016; 102(13): 1009–1016. 27091846 10.1136/heartjnl-2015-308790 PMC4941172

[14] Nieminen T Prättälä R Martelin T , et al. Social capital, health behaviours and health: a population-based associational study. BMC Public Health 2013; 13(1): 1–1. 23805881 10.1186/1471-2458-13-613 PMC3722011

[15] Teo AR Choi H Valenstein M . Social relationships and depression: ten-year follow-up from a nationally representative study. PLoS One 2013; 8(4): e62396. 10.1371/journal.pone.0062396 PMC3640036 23646128

[16] Rönkä AR Taanila A Koiranen M , et al. Associations of deliberate self-harm with loneliness, self-rated health and life satisfaction in adolescence: Northern Finland birth cohort 1986 study. Int J Circumpolar Health 2013; 72(1): 21085. 10.3402/ijch.v72i0.21085 PMC3753134 23984286

[17] Abbott J Lim M Eres R , et al. The impact of loneliness on the health and wellbeing of Australians. InPsych 2018; 40(6).

[18] Ryff C Friedman E Fuller-Rowell T , et al. Varieties of resilience in Midus. Soc Personal Psychol Compass 2012; 6(11): 792–806. 24058379 10.1111/j.1751-9004.2012.00462.x PMC3775270

[19] Verbakel E . Informal caregiving and well-being in Europe: What can ease the negative consequences for caregivers? J Eur Soc Policy 2014; 24(5): 424–441.

[20] Vitaliano PP Zhang J Scanlan JM . Is caregiving hazardous to one’s physical health? A meta-analysis. Psychol Bull 2003; 129(6): 946–972. 14599289 10.1037/0033-2909.129.6.946

[21] Etkind SN Bone AE Gomes B , et al. How many people will need palliative care in 2040? Past trends, future projections and implications for services. BMC Med 2017; 15(1): 1–0. 10.1186/s12916-017-0860-2 PMC5436458 28514961

[22] Hui D Nooruddin Z Didwaniya N , et al. Concepts and definitions for “actively dying,” “end of life,” “terminally ill,” “terminal care,” and “transition of care”: a systematic review. J Pain Symptom Manag 2014; 47(1): 77–89. 10.1016/j.jpainsymman.2013.02.021 PMC3870193 23796586

[23] Creswell JW Clark VL . Designing and conducting mixed methods research. Thousand Oaks, CA: SAGE publications, 2017.

[24] Tashakkori A Creswell JW . Editorial: the new era of mixed methods. J Mix Methods Res 2007; 1(1): 3–7.

[25] Walker S Read S Priest H . Use of reflexivity in a mixed-methods study. Nurse Res 2013; 20(3): 38–43. 10.7748/nr2013.01.20.3.38.c9496 23346778

[26] Braun V Clarke V . Reflecting on reflexive thematic analysis. Qual Res Sport Exerc Health 2019; 11(4): 589–597.

[27] Harrop E Selman L . Bereavement during the covid-19 pandemic in the UK: what do we know so far? Bereavement 2022; 1: 18.

[28] Rentscher KE Zhou X Small BJ , et al. Loneliness and mental health during the COVID-19 pandemic in older breast cancer survivors and noncancer controls. Cancer 2021; 127(19): 3671–3679. 34161601 10.1002/cncr.33687 PMC8419003

[29] Tabaczynski A Bastas D Whitehorn A , et al. Changes in physical activity and associations with quality of life among a global sample of cancer survivors during the COVID-19 pandemic. J Cancer Surviv. Epub ahead of print 26 January 2022. DOI: 10.1007/s11764-021-01156-x PMC8789373 35079964

[30] Marty PK Novotny P Benzo RP . Loneliness and ED visits in chronic obstructive pulmonary disease. Mayo Clin Proc 2019; 3(3): 350–357. 10.1016/j.mayocpiqo.2019.05.002 PMC6713837 31485574

[31] Wang J Mann F Lloyd-Evans B , et al. Associations between loneliness and perceived social support and outcomes of mental health problems: a systematic review. BMC Psychiatry 2018; 18(1): 1–6. 29843662 10.1186/s12888-018-1736-5 PMC5975705

[32] Smith T . “On their own”: social isolation, loneliness and chronic musculoskeletal pain in older adults. Qual Ageing Older Adults 2017; 18: 87–92.

[33] Boston P Bruce A Schreiber R . Existential suffering in the palliative care setting: an integrated literature review. J Pain Symptom Manag 2011; 41(3): 604–618. 10.1016/j.jpainsymman.2010.05.010 21145202

[34] Mako C Galek K Poppito SR . Spiritual pain among patients with advanced cancer in palliative care. J Palliat Med 2006; 9: 1106–1113. 17040148 10.1089/jpm.2006.9.1106

[35] Houtepen R Hendrikx D . Nurses and the virtues of dealing with existential questions in terminal palliative care. Nurs Ethics 2003; 10: 377–387. 12875535 10.1191/0969733003ne620oa

[36] Mihalopoulos C Le LK Chatterton ML , et al. The economic costs of loneliness: a review of cost-of-illness and economic evaluation studies. Soc Psychiatry Psychiatr Epidemiol 2020; 55(7): 823–836. 31119308 10.1007/s00127-019-01733-7

[37] Gerst-Emerson K Jayawardhana J . Loneliness as a public health issue: the impact of loneliness on health care utilization among older adults. Am J Public Health 2015; 105(5): 1013–1019. 25790413 10.2105/AJPH.2014.302427 PMC4386514

[38] James H Crawford GB . Healthcare interpreters and difficult conversations: a survey. BMJ Support Palliat Care. Epub ahead of print 26 July 2021. DOI: 10.1136/bmjspcare-2021-003045 34312185

[39] Barnard A Ganca L . Using communication skills for difficult conversations in palliative care: ‘Suffering is not a question which demands an answer, it is not a problem which demands a solution, it is a mystery which demands a “Presence”. CME 2011; 29(7): 282.

[40] Schoenmakers E . Why and how to talk about loneliness. J Soc Interv Theory Pract 2020; 29(4): 4.

[41] Starfrace S Backman-Hoyle D Vickers B , et al. The value of connectedness: lived experiences of loneliness and isolation in our communities. Aust N Z J Psychiatr 2019; 53(S1): 79–80.

[42] Hasson F Jordan J McKibben L , et al. Challenges for palliative care day services: a focus group study. BMC Palliat Care 2021; 20(1): 1–9. 33435954 10.1186/s12904-020-00699-7 PMC7802306

[43] Bialik K Fry R . Millennial life: How young adulthood today compares with prior generations. Pew Research Center, 2019, p. 14, https://www.pewresearch.org/social-trends/2019/02/14/millennial-life-how-young-adulthood-today-compares-with-prior-generations-2/ (accessed 26 August 2022).

[44] Walshe C Roberts D . Peer support for people with advanced cancer: a systematically constructed scoping review of quantitative and qualitative evidence. Curr Opin Support Palliat Care 2018; 12(3): 308–322. 29979318 10.1097/SPC.0000000000000370

[45] Smith R Drennan V Mackenzie A , et al. Volunteer peer support and befriending for carers of people living with dementia: an exploration of volunteers’ experiences. Health Soc Care Community 2018; 26(2): 158–166. 28736867 10.1111/hsc.12477

[46] Smith R Drennan V Mackenzie A , et al. The impact of befriending and peer support on family carers of people living with dementia: a mixed methods study. Arch Gerontol Geriatr 2018; 76: 188–195. 29547740 10.1016/j.archger.2018.03.005

[47] Bickerdike L Booth A Wilson PM , et al. Social prescribing: less rhetoric and more reality. A systematic review of the evidence. BMJ Open 2017; 7(4): e013384. 10.1136/bmjopen-2016-013384 PMC5558801 28389486

[48] Drinkwater C Wildman J Moffatt S . Social prescribing. BMJ 2019; 364: 1285. 10.1136/bmj.l1285 30923039

[49] McDaid D Bauer A Park AL . Making the economic case for investing in actions to prevent and/or tackle loneliness: a systematic review. London: London School of Economics and Political Science, 2017.

[50] Scottish Government. A Connected Scotland: our strategy for tackling social isolation and loneliness and building stronger social connections, https://www.gov.scot/publications/connected-scotland-strategy-tackling-social-isolation-loneliness-building-stronger-social-connections/documents/ (2018, accessed 2 March 2022).

[51] McConnell T Finucane A Hanna JR , et al. “You’re the first person who’s sat on that sofa in 12 months” Experiences of loneliness among people at the end of life and their carers in Northern Ireland, https://www.mariecurie.org.uk/globalassets/media/documents/policy/policy-publications/2022/experiences-of-loneliness-among-people-at-the-end-of-life-and-their-carers-in-northern-ireland.pdf (2022, accessed 10 February 2022).

