# Supplementary material for: ‘There’s something about admitting that you are lonely’ – prevalence, impact and solutions to loneliness in terminal illness: An explanatory sequential multi-methods study
Source: Palliat Med. 2022 Sep 8;36(10):1483–92. doi: 10.1177/02692163221122269 (PMC9749015; doi:10.1177/02692163221122269)
Supplement: sj-pdf-1-pmj-10.1177_02692163221122269 – Supplemental material for ‘There’s something about admitting that you are lonely’ – prevalence, impact and solutions to loneliness in terminal illness: An explanatory sequential multi-methods study [file sj-pdf-1-pmj-10.1177_02692163221122269.pdf]

Supplementary file 1: Survey questions

Loneliness among people who are terminally ill and their informal carers in Northern Ireland: Perspectives and experiences of health and social care professionals

By completing this survey, you are consenting to participate in this study.

Question	Options
**About you**	
1. Gender	Female<br>Male<br>Other [open text]<br>Prefer not to say
2. Ethnicity	White<br>Mixed or Multiple ethnic groups<br>Asian or Asian British<br>Black, African, Caribbean or Black British<br>Other ethnic group
3. Age group	18-30<br>31-40<br>41-50<br>51-60<br>61+<br>Prefer not to say
4. What is your job role in Marie Curie?	Registered Nurse<br>Healthcare Assistant<br>Patient and family support team<br>Rehab Team
5. Number of years' experience providing palliative care in current or previous role	[Open text]<br>Prefer not to say
**Prevalence of loneliness among Marie Curie patients and their informal carers**	
This study adopts the official definition of loneliness used by the UK Government, i.e.: Loneliness is a subjective, unwelcome feeling of lack or loss of companionship, which happens when there is a mismatch between the quantity and quality of social relationships that we have, and those that we want.<br><br>*An informal carer is anyone providing unpaid care or support. This could be a spouse, parent, child, other relative, neighbour or friend.*	
6. To what extent has the COVID-19 pandemic impacted on the prevalence of loneliness among the patients you care for?	No increase in numbers experiencing loneliness<br>Slight increase in numbers experiencing loneliness<br>Moderate increase in numbers<br>Substantial increase in numbers
7. In your opinion/from your perspective, to what extent has	No increase in numbers<br>Slight increase in numbers

the COVID-19 pandemic impacted on the prevalence of loneliness among their informal carers?	Moderate increase in numbers<br>Substantial increase in numbers
While the previous two questions [6 and 7] have focused on loneliness during the pandemic, the following questions focus on loneliness in general (outside of COVID-19)	
8. In your typical pre-pandemic caseload, have you provided care or support for <u>patients</u> who you believe may have been experiencing loneliness?	Yes<br>No [skips to Q11]<br>Don't know [skips to Q11]
9. What caused you to come to this conclusion? Select as many as appropriate.	The patient told me/indicated that they were experiencing loneliness<br>Informal carer told me/indicated that the patient was experiencing loneliness<br>My own observations about the patients' circumstances<br>Other [open text]
10. In your typical pre-pandemic caseload, what proportion of the patients you cared for or supported do you believe may have been experiencing loneliness?	0-25%<br>26-50%<br>51-75%<br>76-100%<br>Don't know
11. In your typical pre-pandemic caseload, have you supported <u>an informal carer</u> who you believe may have been experiencing loneliness?	Yes<br>No [if also selected 'no' to Q8, skips to end of survey; if selected 'yes' to Q8, skips to Q14]<br>Don't know [if also selected 'no' to Q8, skips to Q16; if selected 'yes' to Q8, skips to Q14]
12. What caused you to come to this conclusion? Select as many as appropriate	An informal carer told me/indicated that they were experiencing loneliness<br>A patient or informal carer told me/indicated that the informal carer was experiencing loneliness<br>My own observations about the informal carer's circumstances<br>Other [open text]
13. In your typical pre-pandemic caseload, what proportion of informal carers do you believe may have been experiencing loneliness?	0-25%<br>26-50%<br>51-75%<br>76-100%<br>Don't know
**Impact of loneliness on Marie Curie patients and their informal carers**	
14. How would you rate the impact of loneliness on the end of life care experiences of the patients you cared for or supported?	▪ **Psychological wellbeing**<br>➢ Low<br>➢ Medium<br>➢ High<br>▪ **Spiritual wellbeing**

<table>
<tr><td></td><td>
➢ Low
➢ Medium
➢ High
▪ Physical wellbeing
➢ Low
➢ Medium
➢ High
▪ Social wellbeing
➢ Low
➢ Medium
➢ High
[open text to provide further information, but not mandatory]
</td></tr>
<tr><td>15. How would you rate the impact of loneliness on the end of life care experiences of the informal carers you supported?</td><td>
▪ Psychological wellbeing
➢ Low
➢ Medium
➢ High
▪ Spiritual wellbeing
➢ Low
➢ Medium
➢ High
▪ Physical wellbeing
➢ Low
➢ Medium
➢ High
▪ Social wellbeing
➢ Low
➢ Medium
➢ High
[open text to provide further information, but not mandatory]
</td></tr>
<tr><td colspan="2">Possible solutions for reducing loneliness among Marie Curie patients and their informal carers</td></tr>
<tr><td>16. Is there anything you do in your current practice to address loneliness among a) your patients and; b) their informal carers?</td><td>[open text]</td></tr>
<tr><td>17. In your opinion, what are the barriers to reducing loneliness among a) terminally ill patients and; b) their informal carers?</td><td>[open text]</td></tr>
<tr><td>18. Is there anything else you would like to tell us about loneliness among terminally ill patients and their informal carers, and how it impacts their care towards end of life?</td><td>[open text]</td></tr>
</table>
